# Capacity to conduct health research among NGOs in Malawi: Diverse strengths, needs and opportunities for development

**DOI:** 10.1371/journal.pone.0198721

**Published:** 2018-07-05

**Authors:** Kate Gooding, James N. Newell, Nick Emmel

**Affiliations:** 1 Malawi-Liverpool-Wellcome Trust Clinical Research Programme, Blantyre, Malawi; 2 Liverpool School of Tropical Medicine, Liverpool, United Kingdom; 3 Nuffield Centre for International Health and Development, University of Leeds, Leeds, United Kingdom; 4 School of Sociology and Social Policy, University of Leeds, Leeds, United Kingdom; University of West London, UNITED KINGDOM

## Abstract

**Background:**

The role of non-governmental organisations (NGOs) in health research has attracted growing attention. NGOs are important service providers and advocates in international health, and conducting research can help NGOs to strengthen these service delivery and advocacy activities. However, capacity to conduct research varies among NGOs. There is currently limited evidence on NGOs’ research capacity that can explain why capacity varies or indicate potential areas for support. We examined NGOs’ capacity to conduct research, identifying factors that affect their access to the funds, time and skills needed to undertake research.

**Methods:**

We examined research capacity through qualitative case studies of three NGOs in Malawi, including one national and two international NGOs. Data were generated through interviews and focus groups with NGO staff, observation of NGO activities, and document reviews.

**Results:**

Availability of funding, skills and time to conduct research varies considerably between the case NGOs. Access to these resources is affected by internal processes such as sources of funding and prioritisation of research, and by the wider environment and external relationships, including the nature of donor support. Constraints include limited ability to apply for research funding, a perception that donors will not support research costs, lack of funding to hire or train research staff, and prioritisation of service delivery over research in funding proposals and staff schedules.

**Conclusion:**

The findings suggest strategies for NGOs and for donors interested in supporting NGOs’ research capacity. Above all, the findings reinforce the importance of initial capacity assessments to identify organisational needs and opportunities. In addition, the need for time and funding as well as skills suggests that strengthening NGOs’ research capacity will often require more than research training.

## Introduction

There is growing interest in the role of non-governmental organisations (NGOs) in international health research [1–3]. Defined as formally constituted organisations that are largely independent from government and aimed at promoting welfare rather than making a profit [4], NGOs are typically known for their work as service providers or advocates [5–7]. However, research has grown as an area of NGO activity [1,8–11], and there have been calls for more NGO involvement in research from both NGOs and the wider research community [2,12–15]. The World Health Organization Strategy on Research for Health, for example, urges governments to promote NGO participation in national health research systems [16]. This interest in NGO involvement in research is based partly on the value of research for strengthening NGOs’ own work, particularly with increased attention to evidence-based practice in development [2,8,9,17–19]. NGO involvement also has potential value for strengthening health research: partly due to their service delivery and advocacy activities, NGOs are sometimes well-placed to identify relevant research questions, access field sites, and disseminate research findings [2,18,20–24]. Those calling for NGO involvement in research suggest NGOs are and should be active throughout this research cycle, including identifying priorities, conducting research, dissemination and using research findings [2,12,13]. In this article, we focus on NGOs conducting research.

To conduct research, NGOs need research capacity. We aim to contribute to the understanding of NGO involvement in research by providing information on their capacity to conduct research, and to the understanding of health research capacity by bringing experience from NGOs. Based on the experience of three NGOs in Malawi, we examine factors within and outside the organisations that affect key elements of capacity to conduct research.

There is currently limited evidence on capacity to conduct research among NGOs. Although an extensive literature examines capacity for health research [25–31], this literature focuses on academic institutes and think tanks, rather than on NGOs where research is an additional function alongside service delivery and advocacy. Within reports on NGO involvement in research, limited capacity to conduct research is frequently noted [2,8,9,18,22,32–35]. However, these reports provide little detail on factors that limit capacity or indication of how and why capacity varies between NGOs. Existing reports also focus largely on international NGOs (INGOs), with less attention to research capacity among NGOs based in developing countries. Supporting NGO involvement in research requires more understanding of current capacity gaps and their causes.

A complete definition of research capacity encompasses the entire research cycle, involving the “ability and resources of individuals, organisations and systems to undertake, communicate and use high quality research” [36]. In line with our focus, we concentrate on ability and resources to conduct research. In conceptualising capacity, we draw on analyses of both research and wider organisational capacity. Both sets of literature emphasise that capacity is multi-faceted, involving tangible elements such as technical skills or material resources, and intangible elements such as leadership and commitment [26,28,31,37–43]. The literature also highlights the need for capacity at individual, organisational and institutional or environmental levels, for example individual skills, effective organisational structures, and enabling funding streams [26,29,31,36,38,41,43,44]. Different elements of capacity and different levels of capacity are interdependent, with interactions between elements such as skills and leadership, and between individual, organisational and environmental levels [26,38]. A further insight is the influence of external relationships on capacity, with power differences between organisations affecting scope for action [28,40,42]. In line with this, discussions of “unleashing” research capacity highlight the significance of externally imposed constraints [26]. Finally, required capacity depends on the task, and different kinds of research need different resources [26,45].

Alongside these conceptual insights about the nature of capacity, the literature indicates specific resources and abilities needed to conduct research. Numerous elements are identified within existing analyses, for example infrastructure, library access, and systems for peer review [29,44]. Three resources consistently discussed are funding, staff time and research skills [25,26,29,31,41,46]. These three resources of funds, time and skills are also frequently noted in discussions of NGO research [2,8,9,18,22,32–35], and they emerged as priorities for the NGOs in our research. Consequently, while recognising that capacity to conduct research involves many more components, including wider organisational abilities such as strategic vision or networking [42], we focus on research funding, staff time and skills to reflect primary concerns in the literature and among our research participants.

NGOs play an important role in Malawi’s health system [47]. The NGO sector has grown since 1994, when Malawi moved to a multi-party system [48]. The number of NGOs is unknown, but one estimate suggests over 500 [49]. Early NGO work focused on service delivery, but with increasing political space, NGO involvement in advocacy increased [48]. Most Malawian NGOs rely on funding from foreign donors, often via INGOs. Following the global recession of the late 2000s and cuts to aid in donor countries, funding declined and became increasingly competitive [50]. The new funding environment also involved increasing donor emphasis on evidence to demonstrate results [50], contributing to a growing interest in conducting research among NGOs in Malawi.

In comparison with other low-income countries, Malawi’s health research sector is strong by some standards. An assessment of health research output based on number of publications ranked Malawi as ninth in the WHO African Region [51]. However, human resources, infrastructure and funding for research all remain limited [47,52–55]. There are efforts to develop research capacity, and programmes such as the internationally-funded Health Research Capacity Strengthening Initiative have contributed to a growth in the number of skilled researchers and stronger institutional structures [47,54,55]. NGOs have been part of some initiatives to strengthen research capacity [55], and they are recognised as a stakeholder within national research policies [53,56].

## Methods

We used comparative case studies, involving in-depth examination of experiences in contrasting settings to enhance explanation and understanding of context [57,58]. We focus on three organisations: a Malawian NGO working on issues affecting women and young people (MALN); the Malawi country office of a large INGO working to strengthen health service delivery (INTA); and the Malawi country office of a medium-sized INGO working in several sectors including health (INTB). Key characteristics at the time of fieldwork are summarised in Table 1, using approximate figures to protect confidentiality.

**Table 1:** Case NGO characteristics.

NGO (pseudonym)	Geographic base and reach	Focus areas	Annual budget	Total staff in Malawi	Research staff and skills	Research experience and focus
MALN	Malawian NGO, working in several districts and with some national programmes.	Women and young people, including HIV and reproductive health.	Over $1 million.	Over 70.	- No staff with dedicated time for research. - Small number of staff with experience of university or NGO research projects, or short-term research training (e.g. one week).	- Research recently included in organisational mission and strategy. - Experience of conducting several studies, mainly for internal use but some aimed at district/national government. - Situation analyses to support specific service delivery projects, broader needs assessments to inform future programmes and policy, and assessments of government service delivery as a basis for advocacy. - Typically fieldwork of a few weeks and combining surveys, interviews and focus groups.
INTA	INGO with a headquarters in a high-income country and working in around 70 countries.	Health service delivery, especially HIV.	Around $15 million in Malawi.	Around 800	- Two full-time research staff. Additional research assistants recruited as needed. - Research staff both have several years of research experience in Northern universities.	International research policy that provides high-level commitment to research. Over 10 years of research experience in Malawi. - Focus on operational research aimed at internal, national and international audiences, including prospective studies to test new interventions and assessments of existing activities. - Designs include multi-year trials, use of existing quantitative datasets, and qualitative studies using interviews and focus groups.
INTB	INGO with a headquarters in a high-income country and working in around 8 countries	Multi-sector approach including work on nutrition, water and sanitation, HIV, malaria and livelihoods.	Around $11 million in Malawi	Around 400	- Research manager whose time is split between research and other programme support. - Research manager has masters-level research training. Several other staff with short-term research experience through previous university or NGO work.	- Research included in country strategy. Growing record of research, primarily for internal or national audiences, with some aimed internationally. - Situation analyses to support specific service delivery projects, assessments of the impacts of NGO and government programmes to inform wider policy and practice, and operational research (e.g. field testing new technologies). Designs vary: typically fieldwork of a few weeks combining surveys, interviews, focus groups and observation, but some longer-term trials.

Selection of NGOs was purposeful [59], based on identifying organisations with specific features that enabled relevant insight. Our focus was NGOs involved in service delivery and advocacy as well as research, not, for example, NGOs such as think tanks. We chose NGOs that provided contrasting organisational contexts, such as international and national structures and varied research experience. They were all NGOs that undertook research and saw conducting research as part of their organisational strategy; this stated commitment to research is not typical of all health NGOs. Case NGOs were initially approached by email, with further discussions about their participation through telephone and face-to-face meetings.

Data were collected during six months’ fieldwork in Malawi followed by email, Skype and telephone conversations. Fieldwork was undertaken by an experienced researcher who previously worked in NGOs (KG). This background contributed to rapport with participants, but familiarity with NGO language and settings also created a risk that aspects of NGO practice might be taken for granted and so unquestioned. This risk was managed through critical discussion with co-authors and other colleagues to provide alternative viewpoints and challenge assumptions. A research diary was also kept throughout fieldwork and analysis to reflect on relationships with participants and how these might affect the data.

In-depth interviews were conducted with 26 staff or former staff in the three NGOs, including staff leading on research, directors and those involved in service delivery and advocacy. Ten repeat interviews were conducted, split between seven staff with particularly extensive knowledge of each NGO’s research capacity. Most interviews lasted 1–1.5 hours and took place in private offices. Semi-structured topic guides were used, tailored to each participant’s role and adapted during fieldwork to pursue emerging themes. Interviews asked broadly about each NGO’s research experience and included specific questions related to capacity, such as strengths and challenges in conducting research, variation in capacity between district or country offices, examples of research that could or could not be taken forward, training or capacity building for research, and support from other organisations or donors. Further insights came from numerous informal conversations with these and other staff.

Focus groups were held with four to five staff in MALN and INTB, lasting around two hours. Participants overlapped with those interviewed, leading to similar findings, but group discussions brought some additional perspectives through interaction and cross-checking between participants.

Focus groups and interview participants were selected based on their involvement in and knowledge of the NGO’s research and to give a diversity of organisational positions. Participants were initially approached by email or in person, depending on logistics and whether they had already met the researcher. No one refused to participate, although work schedules meant some staff could not attend focus groups; views from these staff were sought through interviews instead.

Observation of NGO meetings and activities provided additional information on research processes and organisational contexts. The extent of observation varied between cases depending on logistics such as desk space in NGO offices, transport access, and timing; with MALN over five weeks were spent working from their office, whereas observation was limited to attending organisational research meetings in the INGOs. During some meetings in MALN, observation involved a high degree of participation, with KG actively involved in discussion, but in most cases meetings were attended primarily as an onlooker or guest.

Document review provided background information on each NGO. Relevant material was identified through internet searches and discussion with NGO staff.

To provide information on NGOs’ relationships with other actors and the wider context, and some indication of whether issues raised in case organisations were shared more widely, further interviews were conducted with donors (2), government (2), academics with experience of NGO collaboration (3) and other NGOs (4). These participants were selected based on suggestions from knowledgeable contacts in Malawi and other participants.

Methods are summarised in Table 2.

**Table 2:** Summary of methods.

Method	MALN	INTA	INTB	Wider context
Interviews	- 8 staff across 13 interviews - (3 repeat interviews)	- 5 staff across 6 interviews - (1 repeat interview)	- 13 staff across 16 interviews - (3 repeat interviews)	Donors—2 Government—2 Academics—3 Other NGOs—4
Focus Groups	1 (5 staff)		1 (4 staff)	
Participant observation	Over 5 weeks based in office, informal conversations, frequent organisational and research meetings	Informal conversations, one research meeting	Numerous visits to office, informal conversations, one research meeting	
Document review	Research reports, website materials, organisational strategies	Research reports, website materials, organisational strategies	Research reports, website materials, organisational strategies	

All interviews and focus groups were conducted in English. 39 of the 46 interviews and both focus groups were audio-recorded and transcribed verbatim by KG. Where recording interviews was impractical and during observation, detailed notes were taken and expanded the same day.

Analysis was ongoing throughout fieldwork, using new data to inform future methods [59,60]. More focused analysis involved familiarisation with the data as a whole through re-reading transcripts and field notes from interviews, focus groups and observation, followed by thematic coding of both notes and transcripts by hand and in NVivo. Codes included both deductive and emerging themes, and comprised organisational categories (such as ‘funding and donors’ or ‘research skills’); substantive categories (such as ‘research seen as irrelevant’ or ‘focusing on own work areas’); and more theoretical categories about relationships (such as ‘project funding determining research’) [61]. Matrices and network diagrams were used to explore relationships between themes and variations between cases [61–63], for example to identify factors affecting funding and the effect of funding on other elements of capacity. Initial explanations were refined by looking for conflicting evidence and considering alternative interpretations [59,62,64].

All stages of analysis were undertaken by *KG* in discussion with co-authors. A summary of findings was shared with case NGO participants for their feedback. Rigour was also supported through triangulation (within interviews and focus groups, between interviews, focus groups, observation and document review, and between research participants), through prolonged engagement with each NGO to develop rapport, and through steps previously mentioned such as reflexivity, audio recording and seeking negative cases [59,64–66]. The study was approved by the University of Leeds ethics committee (reference number HSLTLM/11/004) and the National Commission for Science and Technology (RTT/2/20) and Centre for Social Research (CSR/11/11/05) in Malawi. Informed consent was based on discussions with NGO directors at organisational level and with individual participants, alongside provision of information sheets. Consent was verbal because NGO participants saw written procedures as unnecessary and inefficient. Written procedures also risk damaging rapport and hence data quality, and they were impractical given numerous informal conversations [67,68]. Where research involves ongoing interaction with participants, valid consent is often achieved through continual open communication [67].

## Results

The case NGOs vary in their capacity to conduct the quantity and type of research they would like. We describe a range of factors that influence their funding, time and skills to conduct research, the three important elements of capacity highlighted in the introduction. Quotes are drawn from interviews, focus groups and informal discussions during participant observation.

### Funding for research

Availability of funds to conduct research varies considerably between the case NGOs. INTA can easily access funds for multi-year research projects; indeed, lack of funding has not prevented any research: “My experience is, all the ideas we had, they were implemented” (programme director). In contrast, while INTB and MALN both secured funding for several studies, other planned research was prevented by lack of funds.

Whether NGOs have the funding they need to conduct research depends on the costs of planned research, their existing funds, and their ability to secure additional funding. On the first aspect of costs, some studies could be undertaken with minimal expense. For example, data for some INTA studies came from the monitoring database of an existing service delivery programme, so no funds were needed for additional data collection. Costs could also be reduced by collecting data as part of ongoing service delivery projects, for example by conducting interviews in communities during regular project monitoring visits. However, these approaches to research only suit particular research questions, and much research is likely to require additional funding for staff time, logistics or other expenses.

Highlighting the second aspect of NGOs’ existing funds, costs for even minimal data collection can be problematic when NGOs do not have any income that they can allocate to research. This difficulty was emphasised by an MALN project officer, who stressed the centrality of funding in determining whether even small-scale research could be conducted:

Even if it’s just a minor issue that you just want to get a clear understanding of what is happening, you require fuel for a vehicle to take you to that particular area. So the resources are very key.

His concern about the cost of transport to collect data reflects MALN’s lack of flexible funding. MALN depend almost entirely on donor grants allocated to specific projects. These grants are tied to plans agreed in advance and cannot easily be used for other activities such as emerging research needs. Such restrictions stalled one study that MALN originally hoped to fund through a project monitoring budget, as they found this budget line could not be reallocated because “we need to adhere to donor requirements” (project officer). In contrast, research ideas were taken forward more easily some years previously when MALN was receiving core funding from one donor. This core support was more flexible, which meant MALN could spend existing income on research and so undertake research without additional funding.

The value of flexible funds for enabling research is exemplified by INTA, where over 80% of funding comes from public donations rather than donor support for specific projects. Funds are transferred from the international headquarters to the Malawi office based on agreed workplans, which can include research, and the country office can request additional funds for relevant activities as further needs arise: “if you need something and you can justify it, you get it” (Malawi director). INTA is consequently able to finance research internally without seeking additional donor support.

When existing funds cannot cover the costs associated with planned research, availability of funding to conduct research depends on ability to secure additional funding from donors. The next sections examine two options for obtaining this funding: incorporating research budgets within service delivery grants, and applying for separate research grants.

### Including research within service delivery project budgets

Including research budgets within grants for service delivery programmes is a particularly relevant strategy given the potential for operational research offered by NGOs’ service delivery activities. However, a combination of internal concerns and priorities and external relationships and funding structures can limit use of this option by NGOs. This is seen in INTB, where staff rarely include a research budget in the service delivery proposals that they submit to donors. One reason is a concern among NGO staff that including research would make proposals uncompetitive:

When we are doing the proposals we have to balance between how much research-related work can we put in versus the chances of us succeeding. Because most of the funding has come through global competitive bidding, which is not an easy thing to do. And then if you have things like research [in the proposal], that automatically reduces your chances of succeeding. (INTB programme manager)

This concern is partly about the impact of including research on overall project costs. It also reflects a feeling that few donors want to fund research. As stated bluntly by INTB’s research manager, including research budgets in service delivery proposals “would require a significant change of mindset of donors”. In particular, donors are thought to want proposals that “show very clearly what the direct number of beneficiaries would be” (INTB headquarters officer), and staff felt such specific, predictable results were easier to estimate with a service delivery intervention than a research project.

When donors are willing to fund research, incorporating research budgets within service delivery proposals requires funding calls that permit inclusion of research and service delivery within the same grant. This integrated approach was followed by one of INTB’s principal donors, which funded a multi-year project involving both research and service delivery. Staff saw this donor as exceptional: with most donors, “there is more compartmentalisation–this is poverty reduction, this is research, and so on”, but this donor “will mix them”, allowing the combination of research and service delivery (INTB funding advisor).

As these comments suggest, INTB staff acknowledged that interest in funding research varies between donors. Some donors clearly will fund research by NGOs, including as part of service delivery programmes. Indeed, one donor interviewed suggested that “for every programme there should be an element of research”, and that including research in proposals “is seen as a plus”. The interest of some donors in funding research means that NGOs’ ability to access research funds depends in part on establishing relationships with the right donors.

Further, while NGO staff saw some donors as unwilling to fund research, these concerns appear in part to reflect assumptions rather than direct donor feedback. Indeed, MALN staff appeared to criticise donors as reluctant to fund research without actually having asked them for support. In addition, when proposals that include research do not receive funding, this may reflect inadequate research plans rather than donor unwillingness to fund research per se; another donor stated that they regularly fund research by NGOs, but this depends on high quality designs. Similarly, NGO interviewees beyond the main case organisations felt donors would fund research provided “you justify properly” (NGO director). However, the concerns among case NGO staff show that regardless of actual donor willingness to fund research, the perception that including research will disadvantage proposals can discourage NGOs from including research budgets in service delivery plans, and so limit their access to research funding.

While concern about donor reactions is one reason research is omitted from service delivery proposals, the priorities of NGO staff are also significant. Although all the case NGOs include research as part of their organisational strategy, research remains secondary to service delivery. Consequently, “when we are designing programmes or projects, research does not take precedence in the list of what we are going to do” (INTB programme manager). This low prioritisation can mean research is not even considered during proposal writing:

From my experience research doesn’t come out as an activity in any discussion. So [laughs], so it’s difficult to remember it when you are doing budgeting. (INTB programme manager)

When NGO staff do consider including research in project proposals, it may be side-lined in favour of service delivery activities seen as having more immediate impact. This applies particularly when the maximum funding available from donor grants is considered inadequate to meet service delivery needs:

In many cases when we design the project we are working with tight budgets, and the priority is to allocate resources to the deliverables. […] So like the boreholes, when you see boreholes, when people have the animals, when they have the seeds. So you would want to make the maximum investment along those lines rather than spending money on research. (INTB programme manager)

When the total funding available is more flexible, it is easier to include a research budget without compromising the priority of service delivery. This is seen in INTA, where the programme director explained that “we try to put extra resources because we don’t want to draw resources from operations”. INTA share the concern to avoid taking resources from service delivery, but can seek additional funding for research more easily through their international headquarters.

A clear indication from donors that they will fund research can overcome both the limited attention to research among NGO staff and their concerns that including research makes proposals uncompetitive. When INTB have included research in service delivery proposals, this has often followed donor encouragement or indeed requirements for research, rather than INTB proposing research and the donor then agreeing: research budgets are included “where there is more of a push from the donor, from the funder, to show how you’re actually learning from your work” (INTB funding officer). This explicit donor interest either enables or necessitates inclusion of a research budget.

### Securing separate research grants

An alternative approach to securing research funding is through separate research grants. Several conditions affect the NGOs’ ability to access such funding. One constraint is lack of time to prepare high quality bids, particularly when there are no or few research staff. In INTB, preparation of research proposals depends on a busy research manager who also has responsibility for other areas of work, so opportunities are missed if he is unavailable. While relying on one person is difficult, finding time is even harder in NGOs without research staff. Applications also require expertise in research funding, and an INTB funding advisor felt their staff have “very little experience” with such grants. Requirements for a track record of research also affect success: securing research funding “requires you to be well established and you should have a name in order to get that type of money that you want for research” (MALN programme manager). This is a difficult condition to meet for NGOs just starting to undertake research.

A further condition involves availability of capable research partners. As mentioned by MALN and INTB staff, working with specialist research organisations or academics can provide the expertise and credentials needed to apply for funding. External partners are also needed to meet the requirements for independence that apply with some research grants, particularly when proposed research evaluates an NGO’s service delivery. This was noted by the INTB research manager in relation to one potential academic collaboration, where “we couldn't lead as we were the ones being evaluated”. Identifying skilled and interested partners can be challenging in a national context like Malawi where research capacity is limited, particularly given limited links between the academic and NGO sectors; academic interviewees suggested “the academic-civil society relationship is very weak” [health academic]. This situation can mean “finding a good research partner is very difficult” (INTB research manager). For INTB, this shortage of partners meant some potential funding applications were abandoned.

The required time, skills, experience and partnership vary between funding schemes. For example, MALN secured funding from a grant scheme designed to build NGO research capacity. These grants did not require independent partners or previous research experience, and the application process was simpler, reducing the time and expertise needed to develop proposals and so facilitating access to funding.

### Staff time and skills to conduct research

Staff time and skills to conduct research also vary between the NGOs. INTA has two full-time research staff, with additional research assistants recruited as needed. INTB has a research manager, but his time is split between research and other programme support. MALN has no staff with dedicated time for research. Assessment of research skills is complex, but as an indication, MALN’s programme manager described existing skills as “very limited”, INTB’s research manager has masters-level research training, and INTA’s two lead researchers have several years of academic research experience.

As with funding, the required time and skills depend on the planned research. For example, INTA has some large quantitative studies that need statistical knowledge and staff to collect data, whereas INTB’s research is often mixed methods and undertaken by consultants, so requiring some understanding of qualitative and quantitative techniques and experience of managing consultancies. This variation in required time and skills applies throughout the results below, which look first at conditions affecting time for research, then at research skills.

### Time for research

Time to conduct research alongside service delivery was identified as a challenge by many NGO staff. As highlighted by a former MALN manager:

As an NGO, if you are engaging yourself with research, that means you have to balance the project implementation and the research that you are carrying out.

To create time for research, NGOs must either allocate research time within the schedules of programme or other staff, or recruit dedicated research staff. Potential difficulties with the former approach are illustrated by MALN’s experience. Research relies on a busy director and programme staff, and difficulty in fitting research around their other work both limits and delays research. One example comes from a research project where no progress had been made a year after the proposal was developed. The director was occupied with international travel and other activities–“I’ve a lot of backlog of work”—and a project officer asked to lead the research was busy with external meetings and an increased service delivery workload caused by a colleague’s resignation:

Unfortunately there’s been a lot going around, I was in Kenya, I was attending a meeting, I was up and down. [The project officer] left, so she has handed over the project to me…

Several internal and external issues contributed to this delay and affect ability to balance research and service delivery workloads more generally. One issue is prioritisation, something emphasised by the former MALN manager quoted above. MALN create time for research around service delivery when particular studies are considered priorities. However, the generally higher prioritisation of service delivery often means research is postponed.

Another issue is ability to plan and reserve time for research. On a daily basis, reserving time for research is made harder by the responsive nature of MALN’s service delivery, which sometimes involves dealing with urgent incidents in programme districts or requests from local people. Discussing delays with some studies, a staff member explained that:

For an NGO, you can’t be in the office and put up a sign on the door saying ‘please don’t disturb’. That doesn’t exist for a service provider!

Longer-term planning also affects time to conduct research. MALN staff complained that some studies were delayed or incomplete because work on the research was inconsistent and fitted around other activities:

When we do our studies it’s like we are doing an activity that will stop midway and then we will pick it up later on, and then it will stop and then we will pick it up. (MALN programme manager)

Building set periods of time for research into annual workplans could allow NGOs to concentrate on and complete research. However, the ability to allocate particular weeks for research is made harder by dependence on donors. Insecure funding can encourage NGOs to take on multiple projects; indeed, MALN’s director described their projects as “countless”. Balancing numerous projects means different deadlines, funding streams and stakeholders, hindering annual planning and time management [69]. Advance planning is also obstructed by the unpredictability of donor funding. As explained by an MALN staff member, “the problem is that you plan to start in March, you only get the funding in June, and by then you have other things on”. This unpredictability is a particular challenge for MALN because limited core funding to cushion inconsistent donor disbursements means donor delays can derail workplans [70]. Uncertain funding also meant annual plans were partly lists of desired activities rather than a guide to what activities would be conducted and when: MALN logframes noted funding for many activities as “to be identified”. This context makes it harder to effectively reserve time for research around service delivery.

As well as affecting planning, the external funding environment limits time to conduct research by increasing the workloads of managers and service delivery staff. A particular constraint is short-term funding restricted to programme activities without support for full staff costs. Such funding was a concern in MALN, where the director complained that donors want NGOs to deliver activities but “don’t want to contribute to salaries”. Limited funding for salaries means service delivery staff juggle multiple service delivery project activities, reducing time to conduct research. Small, short-term grants also contribute to the constant focus on securing new donor funding. In MALN, much of the director’s week is spent networking with donors to maintain or seek funding, again taking time that could be used for research.

Creating staff positions with dedicated time for research can overcome the challenge of balancing service delivery and research workloads. This was evident in INTB, where staff stressed the importance of their research manager position in allowing time to conduct research alongside the priority of service delivery:

If the research was put under [the project managers], nothing would have happened, because they are so busy with the day to day running. But in this case it’s fantastic, we have the resource, we have [the research manager] who can concentrate on that element. It wouldn’t matter whether the whole month he doesn’t make an input into the [service delivery project], the project will still run because there are full-time [programme staff].

As well as contributing hours themselves, research staff can promote attention to research and so increase its priority in organisational workloads. This was described by an INTB officer based in the headquarters when discussing the greater volume of research in their Malawi country office compared to offices without research staff:

Having that research post means you’ve got someone [whose] main focus is on research, and they’re driving that agenda forward within the programme, and that does make a difference.

However, employing research staff requires funding. INTA can hire staff using their core funding, and in INTB, a donor funds the research manager position. Lacking donor support or core funds, MALN hoped to recruit a research manager but this was “funds permitting, if we have enough resources” (programme manager). Financial inability to hire staff, combined with heavy and unpredictable workloads, makes time to conduct research a significant constraint in MALN.

### Research skills

As with staff time, availability of skills to conduct research is affected by funding. NGOs with more funding to spend on salaries can hire staff with stronger research skills, something highlighted by a former MALN manager:

You already know the difference between local NGOs and international. The international, because they have got access to more resources, they would get the right people to carry out the research, with skills, and they would be able to pay them the way they want. While in MALN as a local NGO, probably that could take time to develop because there’s limited access to resources.

INGOs’ ability to pay higher salaries can enable recruitment and retention of staff with more qualifications and experience, attracting skilled staff away from national NGOs. The former MALN manager is one example: they were among the staff with most research experience, but moved to an INGO.

Funding also affects scope for training to enhance research skills among existing staff. For example, MALN staff discussed the potential value of training by university researchers, but noted that “those guys can support you at any time provided you have the resources, that’s the difficult part” (programme manager). In contrast, INTA’s flexible core funds allow them to hire experienced research staff who mentor others, and to run international research courses and national workshops that build skills among existing staff (as shown in observation of a meeting to plan capacity building for staff new to research).

While funding has a critical influence on availability of skills to conduct research, availability of time and prioritisation of research are also significant. MALN were not fully using existing research skills or exploiting potentially free or low-cost opportunities to develop skills. For example, observation showed that two headquarters staff had basic research skills from previous university work, and could have used these skills more extensively, both to undertake research and mentor others. In contrast, INTB’s research manager actively sought inexpensive ways to develop organisational research skills. The difference was illustrated during fieldwork: MALN asked one of us (KG) to provide research training, but did not allocate staff time for this, whereas INTB requested support and quickly organised a training workshop. The different approaches partly reflect the contribution of INTB’s research manager position: compared with MALN’s busy director, the research manager has more time to identify and pursue research training opportunities, and so to enhance organisational skills to conduct research.

## Discussion

Our findings indicate variations in funds, time and skills to conduct research among NGOs, and internal and external factors that affect availability of these resources. As shown in the introduction, existing literature points to gaps in NGOs’ research capacity (e.g. [2,18,22]). Our findings help to explain both these capacity gaps and variations in research capacity among NGOs. Table 3 summarises the findings, indicating key factors that affect NGOs’ funding, skills and time to conduct research.

**Table 3:** Factors affecting availability of funds, time and skills to conduct research in NGOs.

Do NGOs have adequate capacity to conduct research?		
Key questions		Influencing factors
**Is adequate funding available to conduct research?**	Does research need additional funding?	- Research design (e.g. small scale research using existing data or activities or large multi-year trial). - Existing funds
	Do NGOs have flexible core funds that they can spend on research?	- Split of organisational income between public donations, donor grants linked to particular activities, and core grants from donors.
	Are research budgets included in service delivery grants?	- NGO staff concerns that donors are unwilling to fund research or that including research will make proposals uncompetitive - Budget ceilings for new grants - Whether funding schemes allow inclusion of research alongside service delivery - Prioritisation of service delivery by NGO staff - Donor indications of interest in funding research
	Can NGOs secure research grants?	- Staff time and skills to apply for grants - Track record of successful research - Links to interested and capable research partners - Requirements of potential grants (e.g. level of requires experience or independence)
**Is adequate time available to conduct research?**	How much time is needed?	- Research design (e.g. large project with intensive data collection, small analysis of existing data)
	Do service delivery/advocacy staff or general managers have time for research?	- Prioritisation of research - Staff workloads - Panning and time management - Predictability of donor funding and full support for staff costs (to enable planning and reduce workloads)
	Do NGOs have staff with dedicated time for research?	- Availability of core funds or donor support to hire research staff
**Are adequate skills available to conduct research?**	What research skills are needed?	- Research design (e.g. quantitative or qualitative study)
	Can NGOs hire and retain staff with research skills and experience?	- Adequate funding to pay salaries that attract skilled staff
	Can NGOs train existing staff in research skills?	- Funds for training - Internal staff who can provide training - Recognition and use of low-cost or free opportunities to build skills

Several constraints identified among the case NGOs are indicated in other literature. For example, concerns about time to conduct research alongside service delivery [18,20,22] and about including research budgets in proposals [22] are also reported for other NGOs. Literature from Malawi and elsewhere also discusses the broader processes found to affect research capacity in the case NGOs, including a lack of core funding, limited donor support for staff salaries, unpredictable and short-term grants tied to specific activities, and the move of skilled staff from national to international organisations [39,69–77].

The NGOs’ experiences also reflect more conceptual aspects of the nature of research capacity identified in other contexts, including interdependence between different elements of capacity and between individual, organisational and environmental levels [26,38], the effect of external relationships [40,42], the role of intangible resources [26,28,31,37–42], and fitting capacity to needs [26,45]. In relation to interdependence between elements of capacity, there are interactions between skills, time and funding to conduct research within the case NGOs. For example, securing research grants requires time and research experience, while flexible core funds or donor support enable access to time and skills by allowing NGOs to hire research staff and provide training. Conversely, insecure, small grants make it harder to allocate time for research by increasing service delivery and fundraising workloads and hindering planning. Links between different levels of capacity are also clear, for example the international funding environment affects organisational access to funding for research training, and consequent scope to strengthen individual research skills. These effects of funding highlight the influence of external relationships, particularly with donors. The role of intangible resources is seen in the significance of prioritisation for research capacity, something noted for NGOs [8] and other research organisations [29]. For example, prioritisation of service delivery over research can mean NGO staff do not include research in project proposals, make time for research in their schedules, or use existing research skills. However, increased prioritisation of research would not overcome all resource constraints. For example, when NGOs need to implement contractually obliged activities and cannot afford to hire extra staff, making time for research is harder than when research staff are available. Finally, required skills, time and funding vary between and within the case NGOs depending on research aims and designs. NGOs that conduct multi-year research trials need different types and levels of skills and funding to NGOs interested in short-term qualitative research.

NGOs provide a different organisational context to the universities and think tanks that form the focus of most literature on health research capacity. Comparing the case NGOs’ experiences with this literature suggests some commonality in the constraints faced by NGOs and other kinds of research organisation in developing countries. For example, limited ability to recruit and retain skilled research staff and lack of core funding are also discussed in relation to academic institutes [28,29,78]. The findings also point to differences. For example, NGOs may face challenges of balancing time for research with service delivery, rather than the balance with teaching responsibilities identified in universities [31]. Differences in scope and ambition of research agendas also mean different capacity requirements. Assessments of capacity in universities or other research-focused organisations highlight challenges around adequate numbers of research staff in different disciplines and with different levels of expertise [25,28,31,79]. In NGOs where research agendas are smaller, lower levels of staffing may be considered adequate. For example, INTB staff appreciated having one staff member whose work is partly focused on research.

Our research has limitations. We focused on NGOs that undertake research and that have a stated commitment to research. NGOs that are not conducting any research may face additional capacity constraints. We also focused on three key elements of capacity to conduct research, funding, skills and time. Complete research capacity includes many more components, including skills related to other aspects of the research cycle such as ability to identify research questions, and wider organisational capacities [26,28,31,44]. Finally, while NGO staff emphasised donor reluctance to fund research, the need to protect organisational confidentiality and study timeframes limited scope to verify their concerns through discussion with donors. The donors interviewed were known to be interested in research, and further investigation is needed to understand approaches to funding NGO research among a wider range of donors.

## Conclusions

Our findings have implications for strategies that could support NGO capacity to conduct research. Above all, a first conclusion is the need to tailor capacity development to specific organisational contexts. Particular NGOs’ methods for developing research capacity are sometimes described as a model for other organisations (e.g. [80,81]). However, diverse research approaches, internal conditions and external relationships create differences in required and available capacity and in opportunities for capacity development. Some recommendations for developing NGOs’ research capacity may only be feasible in large organisations with flexible core funding. For example, advice to hire research staff and provide training [18] is more easily followed with adequate flexible funding or donor support. Similarly, recommendations to give staff dedicated time for research and to include research time and budgets in annual plans [18] are more achievable when NGOs can recruit additional staff and when they have secure funding that provides control over annual plans and budgets. Varied starting points among NGOs necessitate capacity assessments, something emphasised for research capacity development more widely [31,41,43].

Second, the importance of multiple components of capacity and links between funding, time and skills to conduct research mean that developing NGO research capacity requires more than skills training, again a message underlined in discussions of organisational and research capacity [28,39,41,82]. Research training courses have demonstrated an impact on production of research by NGO staff [83], but this impact seems likely to be diminished in NGOs without adequate staff time or funding to conduct research.

Third, the influence of prioritisation on availability of funds, time and skills and the influence of research design on requires resources both suggest there are options for NGOs wishing to conduct more research. In particular, NGOs could adapt research designs to suit available capacity: small-scale data collection or use of monitoring records may be feasible without additional resources. NGOs can also explore ways to strengthen capacity within existing resources, for example, there may be staff with research experience who can share skills with others. There may be opportunities for including research budgets within service delivery proposals, or for considering alternative donors if current funders do not support research. NGOs can also consider different models for conducting research, including partnership with other researchers. The potential value of collaboration with academics is often emphasised in discussions of NGOs’ research [2,3,22,35,84,85], and such collaboration is an important strategy for some case NGOs [24]. As INGB’s experience indicates, suitable academic partners can be hard to find, and collaboration faces challenges such as divergent organisational priorities. However, working with academics can help some NGOs that lack internal capacity to conduct research.

Fourth, the significance of external relationships and funding suggests recommendations for donors interested in supporting NGO research capacity. To support access to research funding, particularly for operational research, donors could provide options for including research budgets within service delivery grants, and clearly indicate willingness to fund research so that NGO staff recognise the opportunity and have confidence to apply. Donors could also consider how current funding approaches may constrain capacity. In particular, more predictable, longer-term grants that include support for staff costs might reduce time spent on securing funding and ease staff workloads, freeing up NGOs’ time for research. For some NGOs, a useful area for donor support may be covering salaries for skilled research staff who can undertake research, apply for funding, and develop research skills and prioritisation among other staff.

The feasibility and effectiveness of these strategies could usefully be explored through action research with interested NGOs and donors. Such an approach might strengthen research within participating organisations, and provide further understanding of constraints and enablers to NGO involvement in health research.
